# The Role of Axillary Lymph Node Dissection Width and Radiotherapy in Axillary Vein Pathologies and Psychophysical Outcomes in Breast Cancer

**DOI:** 10.3390/medicina61071212

**Published:** 2025-07-03

**Authors:** Mujdat Turan, Ibrahim Burak Bahcecioglu, Sumeyra Guler, Sevket Baris Morkavuk, Gokhan Giray Akgul, Sebnem Cimen, Elif Ayse Ucar, Ebru Umay, Mehmet Mert Hidiroglu, Yasemin Ozkan, Mutlu Sahin, Kerim Bora Yilmaz

**Affiliations:** 1 Department of General Surgery, Ankara Gulhane Research and Training Hospital, University of Health Sciences, Ankara 06010, Turkey; 2 Department of Surgical Oncology, Gulhane Research and Training Hospital, University of Health Sciences, Ankara 06010, Turkeyguler.sumeyra@yahoo.com (S.G.);; 3 Department of Radiology, Ankara Etlik City Hospital, Ankara 06170, Turkey; 4 Department of Physiotherapy and Rehabilitation, Ankara Etlik City Hospital, Ankara 06170, Turkey; 5 Department of Education, Ankara Gulhane Research and Training Hospital, University of Health Sciences, Ankara 06010, Turkey; 6 Department of General Surgery, Nev Health Group, Bursa 16310, Turkey

**Keywords:** breast cancer, axillary lymph node dissection, axillary vein pathologies, Nottingham Health Profile, DASH questionnaire

## Abstract

*Background and Objectives:* Lymphedema is one of the most important morbid complications of modified radical mastectomy (MRM) surgery. It can cause limb movement restriction and psychosocial deformities in some patients. This study aimed to determine and compare the physiological and pathological changes that develop in the axillary venous structures in patients who underwent axillary lymph node dissection (ALND) and sentinel lymph node biopsy (SLNB). *Materials and Methods:* Patients diagnosed with breast cancer who underwent MRM and breast-conserving surgery (BCS) plus SLNB between 2017 and 2022 were retrospectively examined. The patients’ operation side and contralateral axillary vein diameter and the difference between them, axillary vein flow rate and the difference between them, axillary vein wall thickness and the difference between them, severity of lymphedema, extremity joint restriction examination, and the Nottingham Health Profile (NHP) data were recorded. The relationship of these parameters with the lymph node dissection width and radiotherapy was analyzed. *Results:* Fifty-eight patients in total were included in the study. In the distribution of lymphedema and lymphedema severity according to ALND groups, there is a statistically significant difference (*p* < 0.001). A statistically significant difference was determined in the distribution of the difference in the axillary vein blood flow rate and axillary vein diameter difference between the two arms according to the lymph node dissection groups. In the distribution of physical therapy and rehabilitation scales according to the lymph node dissection groups, a significant difference was found in the disabilities of the arm, shoulder, and hand (DASH), shoulder flexion restriction variables, and NHP sleep variables (all *p* < 0.001). *Conclusions:* This study demonstrated that ALND leads to more pronounced physiological and pathological changes in axillary venous structures—including increased vein wall thickness, altered flow rates, and diameter differences—compared to SLNB combined with breast-conserving surgery. These changes may be attributed to lymphovenous disruption and postoperative edema. Furthermore, radiotherapy appears to contribute to these changes, though to a lesser extent than ALND. Therefore, SLNB followed by radiotherapy may be preferable in eligible patients to reduce postoperative complications such as lymphedema, joint restriction, and sleep disturbances.

## 1. Introduction

Breast cancer is the most commonly diagnosed cancer among women worldwide, representing approximately 30% of all female cancers [1]. It remains a leading cause of cancer-related mortality. Surgical treatment plays a pivotal role in management: Breast-conserving surgery (BCS) is performed in 60–70% of early-stage cases, typically with sentinel lymph node biopsy (SLNB). In developed countries, the use of SLNB rose from around 11% in 1998 to nearly 59% by 2004, and between 2012 and 2013, approximately 90% of clinically node-negative patients undergoing BCS received SLNB [2]. Axillary lymph node dissection (ALND) is generally reserved for cases with confirmed lymph node involvement in stage I–II disease [3].

Modified radical mastectomy (MRM) is still a significant choice preferred by experienced surgeons due to its low morbidity and complication rates in breast cancer treatment. When the literature is reviewed, it is seen that there are postoperative surgical complications (hematoma, infection, seroma, lymphedema, etc.) in 8–26% after MRM [4]. ALND has a significant place in the morbidity-inducing part of these complications. In recent years, especially in early-period breast cancer cases, BCS has come to the fore in treatment plans. BCS is not a treatment method by itself; it is rather a combined method in which regional (radiotherapy) and systemic treatment (chemotherapy) options are used in combination [5]. When compared with MRM, there is no significant difference between early-period recurrence and long-term survival rates of the patients who underwent BCS. This result has led to a tendency towards breast-conserving and oncoplastic breast surgery techniques in the surgical treatment of breast cancer [6]. The minimally invasive approach in breast surgery has also found a place in axillary dissection. With the SLNB technique, surgical morbidity rates induced by ALND have been tried to be decreased [7,8,9].

Although the disruption in the lymphatic system circulation is primarily responsible for the lymphedema developing after MRM, it is known that the venous system plays a role in this disruption. The literature review shows that venous obstruction has been detected in 20% of lymphedema patients [10]. It has been seen that venous pressure increased in the Doppler ultrasonography carried out on the edematous upper extremity. With axillary vascular examination performed with color Doppler ultrasonography, anomalies reaching up to 70% have been determined [11]. Nevertheless, whether axillary venous disease status has an effect on the development mechanism of postmastectomy lymphedema that may develop even a long time after MRM is still controversial [12]. Radiotherapy (RT) is the main culprit in this matter. RT leads to destruction in the nerve and vascular tissue with its ionization effect [13,14]. Although this effect is seen in small-diameter veins, large-diameter veins can also be affected, albeit rarely.

There is no consensus on whether the reason for the lymphedema that develops in the upper extremity following a mastectomy is only the lymph system pathology related to the techniques used in the surgery (leakage, scarring, occlusion, …), or late-onset venous obstruction that develops after the surgery, or it is secondary to postoperative radiotherapy. Accepting these three factors as the primary factors means cutting corners. This is because we argue that, in addition to being an independent factor, the coexistence of these three factors has a synergetic effect on the development of lymphedema. In a study comparing the axillary venous status of patients with and without upper extremity lymphedema after MRM, sonographic differences were determined between the two groups [15]. However, although significant differences have been determined between patients who underwent ALND and those who underwent SLNB in terms of complications, there is no study showing the effect of axillary venous structures on these complications.

This study aimed to determine and compare the physiological and pathological changes that develop in the axillary venous structures in patients who underwent ALND and SLNB. In addition, it also aimed to evaluate the effect of radiotherapy, which is a local adjuvant component of breast-conserving surgery, on these changes.

## 2. Materials and Methods

### 2.1. Study Population

Patients diagnosed with breast cancer who underwent modified radical mastectomy and BCS plus SLNB between 2017 and 2022 in the Surgical Oncology Clinic of Ankara Gulhane Training and Research Hospital and the General Surgery Clinic of Ankara Diskapi Training and Research Hospital were retrospectively examined. Patients who did not complete adjuvant chemotherapy and radiotherapy, in whom no metastasis or local recurrence was detected, and who were followed up, were prospectively evaluated. The patients’ backgrounds were examined, and those who had peripheral vascular diseases and a vasculitis history and were chronic smokers were excluded from the study. After obtaining the required ethical board permissions, the remaining patients were called and informed about the study plan and objective. Patients who consented to participate in the study were included in the study.

### 2.2. Study Design

First of all, ethical permission for the study was obtained from Ankara Diskapi Training and Research Hospital Ethical Board (Date: 19 February 2018 and Approval No: 46/06). Since this was a retrospective study, no formal sample size calculation was performed. All eligible patients treated between 2017 and 2022 who met the inclusion criteria were included in the analysis. In the study plan, the aim was to evaluate all patients by a single radiologist with Doppler ultrasonography. The patients’ operation side and contralateral axillary vein diameter and the difference between them, operation side and contralateral side axillary vein flow rate and the difference between them, operation side and contralateral side axillary vein wall thickness and the difference between them, and intraluminal thrombus, phasic, and continuous blood flow data were recorded. After that, all of them were evaluated by a single physical therapy and rehabilitation specialist. The patients’ presence and severity of lymphedema, extremity joint restriction examination, and the Nottingham Health Profile (NHP) data were recorded. The relationship of these parameters with the lymph node dissection width and radiotherapy was analyzed.

### 2.3. Ultrasound Examination

Before the examination, patients rested for 10 min in a room with the temperature at 25 °C in the supine position. Sonographic examinations were performed by using an Esaote Biomedica MyLab 5 system (Esaote SpA, Genoa, Italy) equipped with a 5–13 MHz linear transducer. All sonographic examinations were performed by the same radiologist who did not have any clinical information about the patients. The patient was situated in the supine position with abduction and flexion of the right elbow at 90° while the left arm was in a relaxed position. The probe was positioned in the axillary region. Firstly, the diameter of the axillary vein was measured in transverse planes. The change in the diameter of the vessel with respiration was evaluated. If there was a change, the wall motion was considered normal. The flow rate of the axillary vein was measured in longitudinal planes. The wall thickness of the axillary vein was measured in transverse planes. Each evaluation was performed thrice, and averages were taken. Noncompressibility of the vein and intraluminal thrombus, which are the findings of venous thrombosis, were evaluated and noted, if any. The flow of the vein was evaluated in the spectral analysis as normal phasic flow (changing with heart pulsations) or pathological continuous flow. Then, the same process was repeated with the left arm.

### 2.4. Lymphedema Examination

The circumference measurements were made at the level of the metacarpophalangeal joint and wrist (proximal ulnar styloid), as well as 10 cm proximal and distal to the lateral epicondyle. Patients with at least a 2 cm difference between the two upper extremities in at least one level were considered to have lymphedema. For severity, the lymphedema was considered mild if the difference between the circumferences of the two arms was <3 cm, moderate if between 3 and 5 cm, and severe if >5 cm. The criteria used to diagnose and classify lymphedema severity were based on established clinical guidelines and previous studies [16,17].

### 2.5. Disability Examination

The disabilities of the arm, shoulder, and hand (DASH) questionnaire is a 30-item questionnaire that checks the ability of a patient to perform certain upper extremity activities. This questionnaire is a self-report questionnaire where patients can rate difficulty and interference with daily life on a 5-point Likert-type scale.

The QuickDASH is an abbreviated version of the original DASH outcome measure. In comparison to the original 30-item DASH outcome measure, the QuickDASH only contains 11 items. It is a questionnaire that measures an individual’s ability to complete tasks, absorb forces, and the severity of symptoms. The QuickDASH tool uses a 5-point Likert-type scale from which the patient can select an appropriate number corresponding to their severity level/function level. The total score to be obtained from the scale is between 0 and 100 points. A higher score indicates greater disability.

### 2.6. The Nottingham Health Profile (NHP)

Designed to measure subjective health status, NHP contains 38 questions grouped into six domains: physical mobility (eight items), social isolation (five items), emotional reactions (nine items), pain (eight items), sleep (five items), and energy (three items). Each question is answered as “yes” or “no” according to whether the symptom is present or absent “in general”. Items in each domain are assigned a weight; the total score for each domain is 100, where a score of 0 indicates good subjective health status and 100 indicates poor subjective health status. 

### 2.7. Statistical Analysis

SPSS v22.0 was employed in the analyses. The compliance of the numerical data in the study with normal distribution was checked with the Kolmogorov–Smirnov test. The median (minimum, maximum) was used in the representation of the descriptive statistics of the variables. Numbers (*n*) and percentages were used for the categorical variables obtained within the scope of the study.

In the comparison of two independent groups, the Student’s *t*-test was used for parametric data, while the Mann–Whitney U-test was employed for nonparametric data. In the analysis of the categorical variables, Pearson‘s chi-squared and Fisher’s exact test were used. Correlation analysis was performed with the Spearman test, while the binary logistic regression test was used in the analysis of multiple variables. To test the significance of the difference between the arithmetic medians of two dependent nonparametric groups, the Wilcoxon signed-rank test was employed. *p* < 0.05 was accepted as statistically significant.

## 3. Results

### 3.1. General Characteristics of the Patients

A total of 96 patients who agreed to participate in the study by signing the consent form were analyzed. Four patients were excluded from the study due to past vasculitis, two due to peripheral vascular disease, and 32 due to smoking history. A total of 58 patients were included in the study. The mean age of the cohort was 56.7 ± 10.8 years. All patients included in the study were female, and the average body mass index (BMI) was 27.9 ± 3.3 kg/m^2^. The tumor was located on the right in 32 patients and on the left in 26 patients. Twenty patients underwent SLNB, and 38 underwent ALND. There was lymph node metastasis in one patient who underwent SLNB and 27 patients who underwent ALND. The mean number of metastatic lymph nodes was 2.62. Lymphatic edema developed in 31 patients, and six of them showed a severe course. In the Doppler ultrasonographic examination, the operation side vein diameter was determined to be 5.7 ± 1.14 mm, and the contralateral vein diameter was 6.20 ± 1.09 mm. The axillary vein blood flow rate was 17.75 ± 6.62 on the operation side, while it was 15.20 ± 6.25 m^3^/s on the contralateral side. When the physical therapy and rehabilitation scales were examined, the mean DASH index value was found to be 15.36. The NHP energy index mean score was 16.39 ± 23.37, the NHP pain index mean score was 16.84 ± 21.14, the NHP sleep index mean value was 14.64 ± 12.44, and the NHP physical ability index mean score was 16.87 ± 18.76. The demographic and clinical characteristics of the patients included in the study are presented in detail in Table 1.

### 3.2. Evaluation of Clinical, Physical, and Psychological Parameters According to Lymphatic Dissection Extent

In the first stage of the study, the patients were divided into two groups: those with SLNB (*n*: 20) and those with ALND (*n*: 38). In terms of baseline characteristics, the two groups were similar in age and BMI. The mean age in the SLNB group was 55.1 ± 10.2 years, while it was 57.8 ± 11.4 years in the ALND group (*p* = 0.278). Likewise, the mean BMI was 27.4 ± 3.1 kg/m^2^ in the SLNB group and 28.2 ± 3.4 kg/m^2^ in the ALND group (*p* = 0.081), indicating no statistically significant difference. In the analysis of the groups according to tumor localization, 40.6% of the patients with left-side tumor localization were in the SLNB group, while 59.4% were in the ALND group (*p* = 0.275). Radiotherapy at 78.1% and chemotherapy at 70.5% rates were applied the most frequently to the patients who underwent ALND (*p* = 0.025 and *p* = 0.161). Intraluminal thrombus was observed only in the ALND group, but the difference was not statistically significant (*p* = 0.197). In the distribution of lymphedema and lymphedema severity according to axillary dissection groups, there is a statistically significant difference (*p* < 0.001 and *p* < 0.001). While 90.3% of the patients who were observed to have lymphedema were those with ALND, 63% of the patients who were observed not to have lymphedema were those with SLNB. Some 75% of the patients with mild lymphedema, 100% of the patients with moderate lymphedema, and 100% of the patients with severe lymphedema were in the ALND group. As expected, the lymph node metastasis and lymph node count were statistically significantly higher in the ALND group (*p* < 0.001 and *p* < 0.001). No statistically significant difference was determined between the operation side and contralateral side axillary vein diameter measurements made in the patients who underwent SLNB and ALND (*p* = 0.204 and *p* = 0.974). However, a statistically significant difference was observed in the distribution of the ipsilateral and contralateral branch axillary vein diameter according to the groups (*p* < 0.001). The median value of diameter difference in the patients with ALND was 0.7 mm, while it was 0.1 mm in the patients with SLNB (Table 2). This difference between the groups was statistically significant, but the intragroup difference change was also statistically significant. In the SLNB group, the median ipsilateral axillary vein diameter was 5.75 mm, and the median contralateral vein diameter was 6.21 mm. This difference in the ALND group was statistically significantly higher. In the patients in the ALND group, the operation side median axillary vein diameter was 5.5 mm, while the contralateral side median axillary vein diameter was 6.20 mm. Similarly, a statistically significant difference was determined in the distribution of the difference in the axillary vein blood flow rate between the two arms according to the lymph node dissection groups (*p* < 0.001). The difference between the operation side and the contralateral side in the SLNB group was 0.8 m^3^/s, while it was 3.45 m^3^/s in the ALND group. The change in the difference between the measurement values of the axillary vein diameter and axillary vein blood flow rate before and after the operation was statistically significant. While calculating this value, the ipsilateral side was accepted as a postoperative result and the contralateral side as a pre-operative value. In the patients who underwent SLNB, the operation side median axillary vein flow rate was 14.2 mm^3^/s, and the contralateral median axillary vein flow rate was 13.5 mm^3^/s. This difference in the ALND group was statistically significantly higher. The operation side median axillary vein flow rate in the patients with ALND was 17.6 mm^3^/s, and the contralateral side median axillary vein flow rate was 14.7 mm^3^/s (Table 3). No statistically significant difference was determined in the distribution of the operation side and contralateral side axillary vein thickness according to the lymph node dissection groups.

In the distribution of physical therapy and rehabilitation scales according to the lymph node dissection groups, a significant difference was found in the DASH and shoulder flexion restriction variables (both *p* < 0.001). While the DASH score was 18.19 in patients who underwent axillary dissection, this value was 4.59 in patients who underwent sentinel lymph node biopsy. The median shoulder flexion restriction was −20 in the ALND group, while it was 0 in the SLNB group. In the distribution of the NHP scales according to the lymph node dissection groups, a significant difference was found only in the NHP sleep subdimension that measures sleep quality. The median NHP sleep score was 17.12 in patients who underwent axillary dissection, while it was 4.56 in patients with lymph node sampling (Table 2).

Among the parameters in which a statistically significant relationship was found, the correlations between the operation side and contralateral side axillary vein diameter difference, the operation side and contralateral side axillary vein blood flow rate difference, shoulder flexion restrictions, DASH, and NHP sleep score and lymph node dissection width were analyzed. A negative and statistically significant correlation was determined between shoulder flexion restriction and lymph node dissection width (rho = −0.558, *p* < 0.001). This correlation was also verified through a regression analysis. Each one-unit increase in the lymphatic dissection count leads to a decrease of −0.840 units in the shoulder flexion restriction (B = −0.840, *p* < 0.001). A positive and statistically significant correlation was found between OS-CS axillary vein diameter difference, OS-CS axillary vein blood flow rate difference, DASH, and NHP sleep variables and lymph node dissection width. Each one-unit increase in the lymph node dissection width leads to a 0.023-unit increase in the OS-CS axillary vein blood flow rate difference, a 0.106-unit increase in the OS-CS axillary vein blood flow rate, a 0.703-unit increase in the DASH scale, and a 0.617-unit increase in the NHP sleep scale (Table 4).

### 3.3. Comparison of Radiotherapy Groups with Clinical, Physical, and Psychological Parameters

In the second stage of the study, we divided the patients into two groups: those who received radiotherapy and those who did not, regardless of lymph node dissection. Baseline demographic characteristics, including age, sex, and body mass index (BMI), were evaluated to ensure homogeneity between the groups. The mean age of the patients in the radiotherapy group was 57.3 ± 10.2 years, while it was 56.0 ± 11.1 years in the nonradiotherapy group (*p* = 0.472). The BMI was also similar between the two groups, with mean values of 28.1 ± 3.2 kg/m^2^ and 27.7 ± 3.4 kg/m^2^, respectively (*p* = 0.608). All patients in both groups were female. These findings indicate that the two cohorts were comparable in terms of baseline demographic parameters, minimizing potential confounding effects of age, sex, and BMI on the vascular and functional outcomes assessed. Then, we analyzed the relationship of radiotherapy with the clinicopathological parameters, physical therapy scales, and the NHP scales. As a result, we found a significant relationship between radiotherapy and lymphedema, lymphedema severity, axillary vein diameter difference between the ipsilateral side and contralateral side, axillary vein blood flow rate difference between the ipsilateral side and contralateral side, and the DASH index (*p* = 0.01, *p* = 0.036, *p* < 0.001, and *p* = 0.008). Of the 29 patients who had lymphedema, 21 (71%) were in the group receiving RT. Some 83% of the patients with mild lymphedema and 69.2% of those with moderate lymphedema were in the RT-receiving group. The operation side and contralateral side median axillary vein diameter difference was 0.7 mm in the patients receiving RT and 0.25 mm in the patients not receiving RT. There was no statistically significant relationship in the distribution of the operation side axillary vein diameter and the contralateral side axillary vein diameter according to the radiotherapy groups (*p* = 0.109 and *p* = 0.502). Similarly, there was a statistically significant difference in the distribution of the operation side and contralateral side axillary vein blood flow rate according to the radiotherapy groups. On the other hand, there was no significant difference in the distribution of the operation side and the contralateral side axillary vein blood flow rate according to the radiotherapy groups (*p* < 0.001, *p* = 0.628, *p* = 0.644) (Table 5).

Univariant and multivariant logistic regression analyses were performed to describe and determine the potential effect of lymphedema, the operation side and contralateral side axillary vein difference, the operation side and contralateral side axillary vein blood flow rate, and the DASH scale on the radiotherapy groups. In the univariant regression analysis, an inverse and statistically significant relationship was found between radiotherapy and lymphedema (OR = 0.241, 95% Cl: 0.08–0.723, B: −1.424, *p* = 0.011). In addition, a positive and statistically significant relationship was found between radiotherapy and OS-CS axillary vein difference, OS-CS axillary vein blood flow rate, and the DASH scale. Radiotherapy administration increases the possibility of an increase in the OS-CS axillary vein diameter difference (OR = 106.65, 95% CI: 9.11–1247.52, *p* < 0.01), an increase in the OS-CS axillary vein blood flow rate difference (OR = 3.405, 95% CI: 1.85–6.24, *p* < 0.01) and an increase in the DASH scale (OR = 1.061, 95% CI: 1.005–1.121, *p* = 0.034). In the multivariate analysis, it was found that radiotherapy did not make a significant contribution to the model in the positive or negative direction on lymphedema, OS-CS axillary vein diameter difference, and the DASH scale. It was determined that only the OS-CS axillary vein blood flow rate difference contributed positively to the model (OR = 281.43, 95% Cl: 1.021–77,548.8, *p* = 0.049). In other words, while radiotherapy is an independent risk factor for each of these factors, only the OS-CS axillary vein blood flow rate difference is affected more by radiotherapy administration in the presence of other factors. The accuracy rate of the model is 79.2% (Table 6).

## 4. Discussion

Axillary lymph node metastasis still remains the most important prognostic factor in breast cancer. It is an important factor in the staging of the disease and the planning of adjuvant treatment. For this reason, ALND is accepted as the primary surgical option by many surgeons. In recent years, BCS plus SLNB has gained increasing popularity among surgeons. What is important here is that the main reason for the trend towards a less invasive method is to try to reduce the physical complications caused by axillary dissection rather than the psychological complications caused by the removal of the breast alone. Lymphedema is one of these complications.

Lymphedema can be detected in 6–30% of ALND patients [18]. Haid et al. reported lymphedema in 3% of patients after SLNB compared to 27% after axillary dissection [19]. Similarly, in a study conducted at the Memorial Sloan Kettering Cancer Center, arm swelling was reported in 3% of patients who underwent SLNB within 5 years after the operation, while this rate was 27% in those who underwent ALND [20]. In our study, 53.4% of the patients had lymphedema. 90.3% of these patients were in the ALND group and 9.7% were in the SLNB group. Importantly, the ALND and SLNB groups did not differ significantly in terms of baseline demographic features such as age (*p* = 0.278) and BMI (*p* = 0.081). These results confirm that the observed differences in vascular alterations and upper extremity dysfunctions between the groups are unlikely to be influenced by demographic confounders and instead reflect the procedural burden of extensive lymph node dissection. In the review published by Petrek, it was emphasized that 77% of lymphedema cases after axillary dissection occurred within the first 3 years [21]. The postoperative evaluation times of the patients included in our study are within this period when lymph circulation cannot be fully repaired by the metabolism.

Although disruption in lymph circulation is primarily responsible for the lymphedema that develops in the upper extremity after breast cancer surgery, there are many studies showing that there are also changes in venous and arterial blood flow in these patients [11,22]. Blood and lymphatic systems are in close interaction with each other [23]. In a cadaveric study conducted by Suami et al., different lymphatic pathways that were never seen before in an 81-year-old woman who underwent axillary dissection 11 years ago and did not receive radiotherapy were revealed. However, no lymphovenous changes were detected in this cadaver [24]. However, Aboul-Enein et al. observed the presence of such shunts on the left arm before lymphedema occurred after mastectomy with lymphangiography [25]. Such abnormal connections, also known as lymphatic shunts between lymphatic vessels and superficial vessels, can trigger changes in venous flow and confirm the increase in flow in the axillary and brachial vessels on the same side as surgery. Abnormal lymphovenous shunts may be related to lymphatic flow occlusion as a result of axillary dissection after breast cancer surgery. However, it may also be associated with hereditary factors such as vascular endothelial growth factor (VEGF), which is the regulatory factor of lymphedema development and lymphatic and blood development. Changes in the genes encoding them may result in failure in vascular development [26].

It was determined that venous pressure increased in the Doppler ultrasonography carried out on the edematous upper extremity following axillary dissection. Differences of up to 70% were detected in axillary vessel examination with color Doppler ultrasonography [11]. In a study, arterial and venous flows in the upper extremities of healthy women and women who developed lymphedema after breast cancer surgery were evaluated, and an increase in arterial blood flow rate and a decrease in venous blood flow were reported in the presence of lymphedema. However, in that study conducted by Montgomery et al., blood flow rate was measured with a tetrapolar impedance device and cardiotachometer [27]. In another study, a positive correlation was noted between lymphedema volume and arterial and venous blood flow rate (as lymphedema volume increased, blood flow rate increased [28]. In the said study, it was emphasized that the blood flow rate of the axillary and brachial arteries and veins was evaluated by Doppler ultrasound, that is, arteries and veins were specifically observed. In our study, measurements related to the axillary vein were performed very precisely by the same radiologist with the help of Doppler ultrasound to prevent homogenization and bias.

Pain et al. evaluated women who underwent mastectomy and observed axillary vein stenosis and flow disorder in both women with lymphedema and those without complications [29]. The authors of the study concluded that the decrease in vein wall movement may be due to axillary dissection, not the presence of lymphedema. In our study, we did not find a statistically significant difference in the distribution of ipsilateral and contralateral side diameter and flow rate according to dissection groups. However, when we evaluate both groups within themselves, the rates of operation side vein diameter and contralateral side vein diameter are statistically lower. The difference is more tangible in the ALND group. This statistical difference explains the damage to the axillary vein wall and the associated narrowing of the vessel diameter as the width of the lymphatic dissection increases.

Studies have reported an increase in blood flow rate in the ipsilateral limb compared to the contralateral side. Matheus et al. evaluated the blood flow in the upper extremities of women who presented without lymphedema after ALND. The results showed a significant increase in arterial and venous blood flow rate in the ipsilateral limb after surgery [30]. Similarly, Valinote et al. evaluated 11 patients with lymphedema and observed that 54.6% and 45.4% of women increased blood flow in the subclavian and axillary veins, respectively, compared to the contralateral upper extremity [31]. In our study, flow rate values in MRM patients were higher than the values obtained from the contralateral side. We found a statistically significant difference in the axillary vein flow rate of both arms. It should also be noted that the overall cohort demonstrated a relatively narrow distribution in age and BMI (mean age 56.7 ± 10.8 years; mean BMI 27.9 ± 3.3 kg/m^2^), and consisted exclusively of female patients. This demographic consistency across the study population limits the potential influence of systemic vascular variability and supports the generalizability of the physiological findings to similar clinical contexts. This difference was greater in the ALND group than in the SLNB group. In the study conducted by Montgomery et al., they found a decrease in flow rate compared to our study. However, according to the laws of physics, the fluid flow rate should be higher in a pipe system with a narrowed lumen diameter. Therefore, we think that the results of our study are compatible with the laws of physics.

It has been observed that radiotherapy applied to the axillary region used in adjuvant treatment after axillary dissection is one of the most important factors that increase the risk of lymphedema [31]. Radiotherapy causes damage to nerve and vascular tissues with its ionization effect [13]. Although this is usually in small vessels, it can rarely lead to complications in large vessels. Radiation-induced fibrosis resulting from venous and lymphatic vessel obstruction and lymphocyte depletion due to increased axillary adipose tissue and local fibrosis after radiotherapy also plays a role in the pathogenesis of lymphedema [32]. In our study, lymphedema, diameter difference, and flow rate difference measurements obtained for the ipsilateral and contralateral sides differed in patients with and without radiotherapy. When these differences were compared with the lymph node dissection width, they were lower in patients who received radiotherapy. Lymphedema was present at a rate of 90.3% in patients undergoing ALND, while this rate was 71% in radiotherapy patients, regardless of the type of operation. While the OS-CS diameter difference was similar in ALND and radiotherapy patients, the flow rate difference was higher in radiotherapy patients compared to ALND patients. In other words, patients who undergo off-label extended lymph node dissection are exposed to the ionization effect of radiotherapy as well as mechanical trauma. In addition, the radiotherapy and nonradiotherapy groups were statistically comparable in terms of age (*p* = 0.472) and BMI (*p* = 0.608). This demographic homogeneity strengthens the attribution of venous structural changes, increased DASH scores, and differences in axillary vein flow rates specifically to radiotherapy exposure, rather than underlying patient characteristics.

As is known, the DASH questionnaire can detect and distinguish positive and/or negative changes in the musculoskeletal system of the upper extremity after previous surgery. Undoubtedly, ALND is one of the major surgical operations of the upper extremity, although it is not a musculoskeletal surgery. This is because the sensory and motor abilities of an extremity do not occur only through the musculoskeletal system. Vascular, neural, and lymphatic elements have a very important role in this regard. In a standard axillary dissection, two major nerves (n. thoracicus longus and dorsalis), major vascular structures (thoracodorsal artery and vein, axillary vein) are preserved, and other structures are resected together with the lymph nodes within the boundaries of dissection. This result may cause a negative effect on sensory and motor sensation in the upper extremity. In the literature, publications examining the relationship between lymph node dissection width and DASH score are quite limited. In their study, Yucel et al. reported that ALND and SLNB operations had no effect on the DASH score [33]. However, since only patients who underwent BCS were included in this study, the number of cases was lower than in our study. In addition, since all patients were administered radiotherapy, the effectiveness of radiotherapy was not examined. In our study, both axillary dissection width and the effectiveness of radiotherapy on the DASH score were examined. We found that both dissection width and radiotherapy had a negative effect on the DASH score.

The Nottingham Health Profile is used to measure overall health-related quality of life. NHP is a 38-item questionnaire that includes the domains of physical mobility, social isolation, emotional reactions, pain, sleep, and energy. It was first used in 1970 [34]. There are many studies in the literature on NHP and its relationship with various diseases. However, we did not encounter a study examining breast cancer and NHP, axillary dissection, NHP, and radiotherapy and NHP results in breast cancer. For this reason, our study is a first. However, as a disadvantage of this, we did not have the opportunity to compare our results with other data and results. In our study, we found that axillary dissection had a negative effect on the NHP Sleep variable compared to SLNB. We attribute this result to the paresthesia effect in the extremity caused by lymphedema developing after dissection. We also found that RT had a negative effect on the NHP Sleep variable, independent of lymphatic dissection. However, this effect was lower than the negative effect caused by axillary dissection.

Our study has some limitations. We did not have the opportunity to evaluate the patients in the pre-operative period. Therefore, we do not have any information about the pre-operative wall thickness, diameter, and blood flow rate of vascular structures. To close this gap, we compared the results of the contralateral axillary vein and operation side axillary vein ultrasonography-Doppler results. Since there was no previous surgical intervention, we considered the data related to the contralateral axillary vein as the pre-operative data of the operation side axillary vein. To minimize our margin of error, we excluded patients with a history of vasculitis and a history of peripheral vascular disease.

## 5. Conclusions

We detected physiological changes in axillary vein wall thickness, blood flow rate, and vessel diameter in patients who underwent ALND or SLNB with the diagnosis of breast cancer. We observed that axillary dissection caused more physiological-pathological changes compared to BCS + SLNB. We think that the differences in these parameters develop as a result of the change in lymphovenous shunts and edema after surgery. Similarly, we found that RT may be an effective factor in newly developing negative physiopathological changes in the axillary vein. These negative changes are lower than ALND. Therefore, we recommend that sentinel lymph node biopsy plus adjuvant radiotherapy may be a more appropriate treatment option instead of axillary dissection in appropriate cases to minimize postoperative lymphedema and its complications (limb movement restriction, sleep disorder).

## Figures and Tables

**Table 1:** Demographic and clinicopathological distribution of all patients.

Age, Years	56.7 ± 10.8
**Sex**	
Female	58 (100%)
Male	0 (0%)
**BMI, kg/m^2^**	27.9 ± 3.3
**Operation width, *n* (%)**	
SLNB	20 (34.5%)
ALND	38 (65.5%)
**Localization, *n* (%)**	
Left	26 (44.8%)
Right	32 (55.2%)
**Radiotherapy, *n* (%)**	
Absent	26 (44.8%)
Present	32 (55.2%)
**Chemotherapy, *n* (%)**	
Absent	14 (24.1%)
Present	44 (75.9%)
**OS intraluminal thrombus, *n* (%)**	
Absent	55 (94.8%)
Present	3 (5.2%)
**OS blood flow, *n* (%)**	
Phasic	51 (87.9%)
Continue	7 (12.1%)
**CS blood flow, *n* (%)**	
Phasic	55 (94.8%)
Continue	3 (5.2%)
**Lymphedema, *n* (%)**	
Absent	27 (46.6%)
Present	31 (53.4%)
**Lymphedema severity, *n* (%)**	
Absent	27 (46.6%)
Mild	12 (20.7%)
Moderate	13 (22.4%)
Severe	6 (10.3%)
**Lymph node dissection, number, mean ± SD, range**	16.12 ± 10.26 (1–42)
**Lymphatic metastasis, *n* (%)**	
Absent	30 (51.7%)
Present	28 (48.3%)
**Metastatic lymph node, number, mean ± SD, range**	2.62 ± 4.94 (0–21)
**OS Diameter, mm, mean ± SD, range**	5.7 ± 1.14 (3.60–8.80)
**CS Diameter, mm, mean ± SD, range**	6.20 ± 1.09 (4.20–9.0)
**OS-CS Diameter difference, mm, mean ± SD, range**	0.49 ± 0.31 (0–1.10)
**OS flow rate, m^3^/s, mean ± SD, range**	17.75 ± 6.62 (7.50–37.40)
**CS flow rate, m^3^/s, mean ± SD, range**	15.20 ± 6.25 (4.50–32.70)
**OS-CS flow rate difference, m^3^/s, mean ± SD, range**	2.55 ± 1.56 (0–5.50)
**Shoulder flexion restriction, mean ± SD, range**	−15.17 ± 16.03 (−60–0)
**OS wall thickness, mm, mean ± SD, range**	0.55 ± 0.11 (0.40–0.79)
**CS wall thickness, mm, mean ± SD, range**	0.56 ± 0.11 (0.40–0.80)
**DASH, mean ± SD, range**	15.36 ± 11.23 (1.45–43.18)
**NHP energy, mean ± SD, range**	16.39 ± 23.37 (0–87)
**NHP pain, mean ± SD, range**	16.84 ± 21.14 (0–88)
**Physical ability, mean ± SD, range**	16.87 ± 18.76 (0–78)
**NHP sleep, mean ± SD, range**	14.64 ± 12.44 (1.8–55.93)

BMI, body mass index; SLNB, sentinel lymph node biopsy; ALND, axillary lymph node dissection; OS, operation side; CS, contralateral side; SD, standard deviation; DASH, disabilities of the arm, shoulder and hand; NHP, Nottingham Health Profile.

**Table 2:** Distribution of demographic and clinicopathological factors according to lymph node dissection groups.

	Number of Patients(%)		
Demographic and Clinicopathological Factors	SLNB Group *n*: 20 (34.5%)	ALND Group *n*: 38 (65.5%)	*p*-Value
**Age, years**	55.1 ± 10.2 (29–72)	57.8 ± 11.4 (21–78)	0.278 ^T^
**Sex, *n* (%)**			not applicable
Female	20 (100%)	38 (100%)	
Male	0 (0%)	0 (0%)	
**BMI, kg/m^2^, median, range**	27.4 ± 3.1	28.2 ± 3.4	0.081 ^T^
**Localization, *n* (%)**			0.275 ^X^2^^
Left	13 (40.6%)	19 (59.4%)	
Right	7 (26.9%)	19 (73.1%)	
**Radiotherapy, *n* (%)**			0.025 ^X^2^^
Absent	13 (50%)	13 (50%)	
Present	7 (21.9%)	25 (78.1%)	
**Chemotherapy, *n* (%)**			0.161 ^X^2^^
Absent	7 (50%)	7 (50%)	
Present	13 (29.5%)	31 (70.5%)	
**OS intraluminal thrombus, *n* (%)**			0.197 ^X^2^^
Absent	20 (36.4%)	35 (63.6%)	
Present	0 (0%)	3 (100%)	
**OS Blood Flow, *n* (%)**			0.231 ^X^2^^
Phasic	19 (37.3%)	32 (62.7%)	
Continue	1 (14.3%)	6 (85.7%)	
**CS blood flow, *n* (%)**			0.966 ^X^2^^
Phasic	19 (34.5%)	36 (65.5%)	
Continue	1 (33.3%)	2 (66.7%)	
**Lymphedema** **, *n* (%)**			**<0.001** ^X^2^^
Absent	17 (63%)	10 (37%)	
Present	3 (9.7%)	28 (90.3%)	
**Lymphedema severity** **, *n* (%)**			**<0.001** ^X^2^^
Absent	17 (63%)	10 (37%)	
Mild	3 (25%)	9 (75%)	
Moderate	0 (0%)	13 (100%)	
Severe	0 (0%)	6 (100%)	
**Lymph node dissection, number, median, range**	5 (1–11)	20.5 (9–42)	**<0.001** ^U^
**Lymphatic metastasis, *n* (%)**			**<0.001** ^X^2^^
Absent	19 (63.3%)	11 (36.7%)	
Present	1 (3.6%)	27 (96.4%)	
**Metastatic lymph node, number, median, range**	0 (0–1)	1 (0–21)	**<0.001** ^U^
**OS diameter, mm, median, range**	5.75 (4.50–8.80)	5.55 (3.60–8)	0.204 ^U^
**CS diameter, mm, mean ± SD**	6.21 ± 1.23	6.20 ± 1.03	0.974 ^T^
**OS-CS diameter difference, mm, median, range**	0.1 (0–0.4)	0.7 (0.3–1.10)	**<0.001** ^U^
**OS flow rate, m^3^/s, median, range**	14.2 (7.5–24)	17.6 (9.5–37.4)	**0.004** ^U^
**CS flow rate, m^3^/s, median, range**	13.5 (7–22)	14.7 (4.5–32.7)	0.143 ^U^
**OS-CS flow rate difference, m^3^/s, median, range**	0.8 (0–3)	3.45 (1.5–5.5)	**<0.001** ^U^
**Shoulder flexion restriction, median, range**	0 (−10–0)	−20 (−60–0)	**<0.001** ^U^
**OS wall thickness, mm, median, range**	0.5 (0.4–0.78)	0.56 (0.4–0.79)	0.374 ^U^
**CS wall thickness, mm, median, range**	0.5 (0.4–0.8)	0.6 (0.4–0.8)	0.318 ^U^
**DASH, median, range**	4.59 (1.45–9.52)	18.19 (8.4–43.18)	**<0.001** ^U^
**NHP** **energy, median, range**	0 (0–87)	0 (0–66)	0.713 ^U^
**NHP** **pain, median, range**	7.39 (0–88)	9.47 (0–62.5)	0.557 ^U^
**Physical ability, median, range**	11.54 (0–78)	12.5 (0–66.09)	0.293 ^U^
**NHP** **sleep, median, range**	4.56 (1.8–12)	17.12 (2.99–55.93)	**<0.001** ^U^

SLNB, sentinel lymph node biopsy; ALND, axillary lymph node dissection; BMI, body mass index; OS, operation side; CS, contralateral side; SD, standard deviation; DASH, disabilities of the arm, shoulder and hand; NHP, Nottingham Health Profile; X^2^: Pearson’s chi-squared test, T: Student’s *t*-test; U, Mann–Whitney U-test.

**Table 3:** Pre-operation and postoperation analysis of axillary vein diameter and flow rate.

Variables	Groups	*n*	Mean ± SD Median (Range)	Test Statistics	*p*-Value
Axillary vein diameter for SLNB performed patients, mm	Operation Side	20	5.75 (4.50–8.80)	−3.893	<0.01 ^Z^
	Contralateral Side	20	6.21 ± 1.23		
Axillary vein diameter for ALND performed patients, mm	Operation Side	38	5.55 (3.60–8)	−5.387	<0.01 ^Z^
	Contralateral Side	38	6.20 ± 1.03		
Axillary vein flow rate for SLNB performed patients, m^3^/s	Operation Side	20	14.2 (7.5–24)	−3.854	<0.01 ^Z^
	Contralateral Side	20	13.5 (7–22)		
Axillary vein flow rate for ALND performed patients, m^3^/s	Operation Side	38	17.6 (9.5–37.4)	−5.379	<0.01 ^Z^
	Contralateral Side	38	14.7 (4.5–32.7)		

SLNB, sentinel lymph node biopsy; ALND, axillary lymph node dissection; SD, standard deviation; Z: Wilcoxon signed-rank test.

**Table 4:** Correlation and regression analysis of OS-CS diameter difference, OS-CS flow rate difference, shoulder flexion restriction, DASH, NHP sleep parameters, and between lymph node dissection width.

Correlation of Number of Lymph Node Dissection Number					
Clinicopathological Factors		*n*		rho	*p*-Value
1-OS-CS diameter difference		58		0.770	**<0.001**
2-OS-CS flow rate difference		58		0.710	**<0.001**
3-Shoulder flexion restriction		58		−0.558	**<0.001**
4-DASH		58		0.749	**<0.001**
5-NHP sleep		58		0.583	**<0.001**
**Regression**					
**Dependent Variables**	**Independent Variable**	**B**	**95% Cl for B**	**t**	***p*** **-Value**
1-OS-CS diameter difference	Number of lymph node dissections	0.023	0.017–0.028	8.347	**<0.001**
2-OS-CS flow rate difference		0.106	0.076–0.135	7.143	**<0.001**
3-Shoulder flexion restriction		−0.840	−1.192–0.487	−4.769	**<0.001**
4-DASH		0.703	0.478–0.928	6.268	**<0.001**
5-NHP sleep		0.617	0.338–0.897	4.425	**<0.001**

OS, operation side; CS, contralateral side; DASH, disabilities of the arm, shoulder and hand; NHP, Nottingham Health Profile.

**Table 5:** Distribution of demographic and clinicopathological factors according to radiotherapy groups.

	Number of Patients (%)		
Demographic and Clinicopathological Factors	Radiotherapy (−) (26 Patients 44.8%)	Radiotherapy (+) (32 Patients 55.2%)	*p*-Value
**Age, years**	56.0 ± 11.1	57.3 ± 10.2	0.472 ^T^
**BMI, kg/m^2^, median, range**	27.7 ± 3.4	28.1 ± 3.2	0.608 ^T^
**Sex, *n* (%)**			not applicable
Female	26 (100%)	32 (100%)	
Male	0 (0%)	0 (0%)	
**Localization, *n* (%)**			0.159 ^X^2^^
Left	17 (53.1%)	15 (46.9%)	
Right	9 (34.6%)	17 (65.4%)	
**OS intraluminal thrombus, *n* (%)**			0.109 ^X^2^^
Absent	26 (47.3%)	29 (52.7%)	
Present	0 (0%)	3 (100%)	
**OS blood flow, *n* (%)**			0.083 ^X^2^^
Phasic	25 (49%)	26 (51%)	
Continue	1 (14.3%)	6 (85.7%)	
**CS blood flow, *n* (%)**			0.966 ^X^2^^
Phasic	26 (47.3%)	29 (52.7%)	
Continue	0 (0%)	3 (100%)	
**Lymphedema** **, *n* (%)**			**0.01** ^X^2^^
Absent	17 (63%)	10 (37%)	
Present	9 (29%)	22 (71%)	
**Lymphedema severity** **, *n* (%)**			**0.036** ^X^2^^
Absent	17 (63%)	10 (37%)	
Mild	2 (16.%7)	10 (83.3%)	
Moderate	4 (30.8%)	9 (69.2%)	
Severe	3 (50%)	3 (50%)	
**OS diameter, mm, median, range**	5.9 (4.4–8.8)	5.5 (3.6–7.9)	0.109 ^U^
**CS diameter, mm, mean ± SD**	6.31 ± 1.21	6.11 ± 0.99	0.502 ^T^
**OS-CS diameter difference, mm, median, range**	0.25 (0–0.8)	0.7 (0.1–1.10)	**<0.001** ^U^
**OS flow rate, m^3^/s, median, range**	16.5 (7.5–33.5)	16.05 (9.5–37.4)	0.628 ^U^
**CS flow rate, m^3^/s, median, range**	14.75 (7–31)	13.2 (4.5–32.7)	0.644 ^U^
**OS-CS flow rate difference, m^3^/s, median, range**	1.25 (0–3)	3.65 (0.5–5.5)	**<0.001** ^U^
**Shoulder flexion restriction, median, range**	−5 (−55–0)	−20 (−60–0)	0.088 ^U^
**OS wall thickness, mm, median, range**	0.53 (0.4–0.8)	0.53 (0.4–0.79)	0.436 ^U^
**CS wall thickness, mm, median, range**	0.53 (0.4–0.8)	0.55 (0.4–0.8)	0.555 ^U^
**DASH, median, range**	8.27 (1.45–43.18)	17.12 (1.67–43.18)	**0.008** ^U^
**NHP energy, median, range**	12 (0–87)	0 (0–66)	0.124 ^U^
**NHP pain, median, range**	9.47 (0–88)	7.39 (0–78.13)	0.804 ^U^
**Physical ability, median, range**	11.54 (0–78)	12.5 (0–62.5)	0.331 ^U^
**NHP sleep, median, range**	6.57 (1.8–55.93)	13.2 (2.99–38.9)	0.052 ^U^

BMI, body mass index; SLNB, sentinel lymph node biopsy; ALND, axillary lymph node dissection; OS, operation side; CS, contralateral side; SD, standard deviation; DASH, disabilities of the arm, shoulder and hand; NHP, Nottingham Health Profile; X^2^: Pearson’s chi-squared test, T: Student’s *t*-test; U, Mann–Whitney U-test.

**Table 6:** Univariant and multivariant analyses of risk factors for radiotherapy in breast cancer patients.

Characteristics	Univariate Analysis			Multivariate Analysis		
	B	OR (95% Cl)	*p*-Value	B	OR (95% Cl)	*p*-Value
**Lymphedema**	−1.424	0.241 (0.08–0.723)	**0.011**	−0.608	0.544 (0.092–3.21)	0.502
**OS-CS diameter difference**	4.670	106.65 (9.11–1247.52)	**<0.001**	−22.09	0 (0–297.76)	0.119
**OS-CS flow rate difference**	1.225	3.405 (1.85–6.24)	**<0.001**	5.640	281.43 (1.021–77,545.8)	**0.049**
**DASH**	0.059	1.061 (1.005–1.121)	**0.034**	−0.011	0.989 (0.896–1.092)	0.825

OS, operation side; CS, contralateral side; DASH, disabilities of the arm, shoulder and hand.

## Data Availability

The data sets used and/or analyzed during the current study are available from the corresponding author on reasonable request.

## Abbreviations

MRM, modified radical mastectomy; BCS, breast-conserving surgery; RT, radiotherapy; ALND, axillary lymph node dissection; SLNB, sentinel lymph node biopsy; DASH, disabilities of the arm, shoulder and hand; NHP, Nottingham Health Profile; USG, ultrasonography; OS, operation side; CS, contralateral side.
